# Body Composition Differences Across Ranks and Sexes of Competitive Swimmers: A Cross‐Sectional Study

**DOI:** 10.1002/hsr2.73375

**Published:** 2026-10-10

**Authors:** Mario Mauro, Federica Moro, Vittorio Coloretti, Pietro Bosetto, Matteo Cortesi, Stefania Toselli

**Affiliations:** ^1^ Department for Life Quality Studies University of Bologna Rimini Italy

**Keywords:** health, nutrition, performance, physiology, sports

## Abstract

**Background and Aims:**

Body composition (BC) plays a key role in assessing athletes' health. However, many factors, including sex and performance rank, may influence BC and should be considered when assessing it. Moreover, BC is estimated indirectly, and body volume (BV) represents a key component of densitometric assessment. This investigation aimed to compare swimmers' BC across sexes and performance ranks, compare methods for BF estimation, and develop a model for predicting BV.

**Methods:**

This was a cross‐sectional study. Participants underwent air‐displacement plethysmography (ADP), anthropometric measurements, and bioimpedance analysis (BIA) according to standardized procedures. Sixty‐four competitive (13.6 ± 1.9 training hours per week) swimmers (17.8 ± 4.4 years, 56.3% male; 13.6 ± 1.9 training hours per week) were enrolled from the national competitive list. They were stratified by sex and classified into three groups according to performance rank (Best World Aquatic score, BWA). A two‐way ANCOVA (sex and rank), adjusted for age, was used to compare BC variables. A bootstrap model‐averaging approach was used to select a linear regression model for predicting BV.

**Results:**

Males showed higher BV, total body water (TBW), and fat‐free mass (FFM) than females. Most rank‐related differences were attenuated after adjustment for age; however, BWA showed a strong positive correlation with limb girths and FFM (Pearson rho > 0.5). BF estimated using specific cut‐offs exhibited the lowest error propagation (reduction of 6.42%). The best model was selected with a 18.81% frequency (adjusted *R* ^2^ = 0.981; RMSE = 1.541), and the variables that showed an adequate bootstrap inclusion frequency (> 60%) were stature (*β* = 0.342), stretched arm (*β* = 0.905), hip (*β* = 0.117), thigh (*β* = 0.659) and calf (*β* = 0.508) girths, with a mean relative conditional bias of 1.79%.

**Conclusion:**

The proposed BF estimation method showed lower error propagation in this sample, while the anthropometric model provided an accurate internal prediction of BV. These findings support the use of predicted BV for subsequent BF estimation using specific cut‐offs, although external validation of the BV prediction model is required.

## 1 Introduction

Human physical performance and body composition (BC) represent two sub‐fields of sports physiology with a long history. The advent of radiology in medical applications enabled investigators to define the atomic and molecular properties of the human body [1] and to develop deterministic mathematical models for assessing BC [2]. Probabilistic theories emerged in biological sciences to account for population variability, and all modern in vivo methods introduce quantifiable errors [3]. BC estimation error is randomly distributed across populations and is expected to decrease as sample homogeneity or the number of measurable components increases [4].

Over the last decades, the application of sophisticated methodologies has enabled clinicians to classify the quantities and distributions of body components in athletes, at different levels of analysis [5, 6]. To date, neutron activation analysis (NAA) remains the best method for determining the chemical composition of the body [7], while the 4‐C (components) model is considered the most reliable clinical assessment [8, 9]. However, it has several limitations due to its cost and the need for numerous operators [10]. Consequently, the 2‐C model is widely used to distinguish body fat and fat‐free mass by measuring body volume (BV) through simpler applications [11].

However, sex, race, and phenotypic traits contribute to human heterogeneity [12]. Biological variability is lower in athletes than in the general population, especially when they are engaged in the same sport [6]. For example, when clustered by sex and experience, basketball players showed similar body composition [13], but may differ from athletes with a different background [14]. Thus, human body traits tend to be more homogeneous among athletes engaged in the same sport and with similar training experience.

Several somatic or biological parameters remain of interest, and researchers have provided long‐term validation, enabling more versatile assessments. Although the development of mathematical models to reduce error propagation remains underexplored [13], methods derived from regression analysis have favored a practical, global approach to BC assessment [15]. While many predictive models are available to estimate body fat (BF), few account for specific sports characteristics [16]. In this context, anthropometry is one of the most debated practical sciences [11].

However, anthropometric assessments may be affected by intra‐ and inter‐observer variability [17], and many investigators have debated the utility of bioimpedance analysis (BIA) as a user‐friendly method [18]. BIA is an observer‐independent method that relies on the strong relationship between body fluids and fat‐free tissues [19]. Both methods have received significant attention due to their lower costs, reduced risks, and easier applicability, especially in sports settings [20].

Previous investigators have described the BC of elite male and female adolescent [21] and adult [22] swimmers, but comparisons across competitive ranks within the same sport and using specific assessment methods are still lacking. The present study, therefore, addresses three aims: (i) to examine differences in athletes' physical traits according to rank and sex; (ii) to compare existing methods for BC estimation; and (iii) to develop a novel model for predicting BV.

## 2 Methods

### 2.1 Study Design

The study was conducted in accordance with the STROBE guidelines for cross‐sectional studies [23].

This study used a cross‐sectional design and was conducted at the University of Bologna Sports Sciences laboratory from November 2024 to April 2025. The experimental room temperature ranged from 19.0°C to 22°C, with 40%–60% humidity. Participant recruitment was conducted with the support of trained national swimming coaches, who identified eligible local athletes. The principal investigator informed parents or guardians of minor participants and adult participants of all experimental procedures and risks, and obtained written consent from them. In addition, adolescent participants provided a written assent. The study was conducted in accordance with the Declaration of Helsinki and approved by the University Bioethics Committee (n. 25027). The final dataset does not contain any identifiable information. The dataset is original and has not been used for previous investigations.

### 2.2 Sample

Study inclusion criteria were (a) inclusion in the International Swimming Federation ranking (World Aquatics Points) based on the competitive swimming strokes (freestyle, backstroke, breaststroke, and butterfly) and race distances (50, 100, 200, and 400 m), (b) at least 4 years of competitive swimming with more than 4 km per week, (c) being younger than 35 years. Participants were excluded from the study if they presented any physical condition affecting BC or were unable to meet the experimental conditions.

Sex and Best World Aquatics score (BWA) percentiles (< 25th percentile: low rank; 25th–75th percentile: mid rank; > 75th percentile: high rank) were used to stratify the sample, and six clusters were analyzed:

1. low‐ranked females (BWA < 482.4)
2. mid‐ranked females (482.4 ≤ BWA ≤ 685.2)
3. high‐ranked females (BWA > 685.2)
4. low‐ranked males (BWA < 553.6)
5. mid‐ranked males (553.6 ≤ BWA ≤ 720.3)
6. high‐ranked males (BWA > 720.3)

Sixty‐four participants met the inclusion criteria, with a mean age of 17.83 ± 4.4 years (72.3% of life spent swimming) and 13.6 ± 1.9 h of training per week.

The sample size was estimated a priori based on previous results [13]:

− Anthropometric models: statistical power of 0.8 with a type I error of 5% for a multiple linear regression F test for partial correlation, with 12 tested covariates (stature, girths, and skinfold thicknesses, except for abs and suprailiac due to missing values) and two control covariates (sex and rank), R^2^ difference of 1.2% (0.969–0.957), delta of 0.387.

The required sample size was 64 participants.

### 2.3 Anthropometry

Stature was measured using a validated stadiometer (Health o‐Meter PORTROD, 9500 West 55th Street, McCook, IL, USA) with a precision of 1 mm, according to Lohman et al. [24]. Girths and skinfold thicknesses were measured at standardized body sites on the right side [24]. Girths were measured once at each body site with a non‐elastic tape (Seca 201, Hammer Steindamm, Hamburg, Germany) for the arm, thigh, and calf. In contrast, skinfold thicknesses were measured twice with a validated caliper (Lange, Beta Technology, Santa Cruz, CA, USA) at the biceps, triceps, subscapular, suprailiac, supra‐spinal, abdominal, thigh, medial calf, and lateral calf. The mean values were used for analysis.

### 2.4 Bio‐Impedance Analysis (BIA)

Total body tissue resistance (R) and reactance (Xc) were measured using a hand‐to‐foot BIA analyzer (BIVA PRO, Akern, Florence, Italy) according to validated standards [25]. Participants were asked to comply with the pre‐test conditions, including 4 h of fasting, 12 h of rest, and at least 24 h of alcohol abstinence. R and Xc were measured twice, and the mean values were used for analysis. The phase angle (PhA) was computed as suggested by Piccoli and colleagues [25]. Moreover, R and Xc were adjusted for participants' stature (in meters; RH and XH).

The total body water (TBW) was estimated using the method of Matias and colleagues [18].

### 2.5 Air Displacement Plethysmography (ADP)

Both body mass (BM) and BV were measured using the Bod Pod GS‐X system (Cosmed, Rome, Italy). The ADP was calibrated at the beginning of each evaluation day, at least 1 h before the first test, according to the manufacturer's instructions. The weight balance system operated with a precision of 0.01 kg and an error of less than 0.1%. An automatic calibration consisting of six runs was performed, with an error of ~0.1%, and the chamber volume was measured with an error of ~0.01%. Each participant was tested twice, and a further test was required when the system detected a difference between the first two evaluations exceeding 150 mL. Each swimmer was asked to wear a stretch nylon swimsuit and a swimming cap and to maintain a standardized posture during the evaluation. Crapo and collaborators' method estimated the volume of thoracic gas (VTG [26];. The surface area artifact (SAA) was calculated on Du Bois and Du Bois [27]. Dempster and Aitken's formula [28] finally computed the BV. The final body density (*d*) was then obtained as $\mathrm{BM}/\bar{\mathrm{BV}}\left[\frac{\mathrm{kg}}{L}\right]$, where $\bar{\mathrm{BV}}$ represented the mean of detected BVs.

### 2.6 Body Composition

The following models were used to estimate BF and fat‐free mass (FFM), according to previous results [13]:

2‐C Brozek et al. [29]:

(1) $$\%\mathrm{BF}=(\frac{4.57}{d}-4.142)*100$$

2‐C Siri [2]:

(2) $$\%\mathrm{BF}=(\frac{4.95}{d}-4.5)*100$$

3‐C Siri [2]:

(3) %BF=(2.118∗BV)−(0.78∗TBW)−(1.351∗BM)

2‐C Lohman [17]:

(4) $$\%\mathrm{BF}=(\frac{C_{1}}{d}-C_{2})*100$$

where $C_{1}$ and $C_{2}$ are constants based on age.

The higher level of BF ($f_{1}$) and FFM ($f_{0}$) and their relative densities ($d_{1}$ and $d_{0}$) has been recorded and then applied to customize standards for swimmers, into the general formula:

(5) $$\mathrm{BF}=\frac{d_{1}d_{0}}{d}\;\left(\frac{f_{1\;}-f_{0}}{d_{0}-d_{1}}\right)-\frac{d_{1}f_{1}-d_{0}f_{0}}{d_{0}-d_{1}}$$

where $d_{0}$ (density of FFM) was computed by removing from BV the volume associated with fat mass, obtained as BM/$d_{1}$ where $d_{1}$ was constant at 0.9007 kg/L, and by dividing the FFM by fat‐free volume, obtained by subtracting fat volume from as BV.

Two different models were proposed: the new model I sets *f*_0_ (formula 5) to the lowest measured fat percentage, whereas the new model II sets *f*_0_ = 0, as suggested by Siri [2]*. d*_0_ and *d*_1_ were selected as the highest and lowest values among the other models (2‐C Brozek, 2‐C Lohman, 2‐C Siri, and 3‐C Siri).

In addition, the uncertainty propagation was computed as follows:

(6) $$\sigma_{f}=\sqrt{a^{2}\sigma_{\mathrm{BV}}^{2}+b^{2}\sigma_{\mathrm{BM}}^{2}-2\mathrm{ab}\sigma_{\mathrm{BV}\mathrm{BM}}}$$

where *a* and *b* are scalars, *σ* is the standard deviation of the parameter, and *σ*_BV BM_ is the covariance of measured parameters. The resulting error was then divided by BM to obtain the desired percentage.

### 2.7 Statistical Analysis

Central tendency and dispersion of the variables were quantified using the mean, standard deviation, and coefficient of variation (CV), while skewness was used to describe the distribution's symmetry. The technical error of repeated measurements (TEM) was computed as the $\sqrt{\sum {∆}^{2}/2n}$ and its relative effect as (TEM/mean) *100, while the coefficient of reliability was computed as [1−(TEM^2^/SD^2^)] [30]. The intraclass correlation coefficient (ICC) and its 95% confidence interval (CI) were estimated to quantify measurement precision. Linear correlation was assessed by the Pearson product‐moment coefficient (*ρ)*. The distributions of variables and residuals were checked graphically and using the Kolmogorov–Smirnov, Cramér‐von Mises, and Anderson–Darling tests. A two‐way ANCOVA was used to compare anthropometric variables across sex and rank, adjusted for age, and to examine both interaction and main effects. Post hoc contrasts were then computed using Bonferroni adjustment. A one‐way ANCOVA was used to compare body fat models, adjusted for age and sex.

For variable selection, bootstrap model averaging was used. Seventy percent of the records were assigned to the training set, and 30% to the test set [31]. One hundred bootstrap samples with replacement were generated, applying the LASSO (least angle selection and shrinkage operator) estimator [32] with the AICC criterion. An event‐per‐variable (EPV) ratio ≥ 10 was considered recommended for the global model [33]. The bootstrap inclusion frequencies (BIF) were recorded, and only variables with BIF values greater than 60% were selected [33]. The model frequencies were used to select the best model, and the average predictions were compared between the global model and the selected one. For stability investigations and sensitivity analyses, the root mean square difference (RMSD) of the bootstrap estimates between the global and selected models, their ratio, and the relative conditional bias were computed [33]. In addition, the model selection frequencies were used as an indicator for model stability. All anthropometric parameters, as well as sex and rank, were treated as regressors. For the selected model's internal assessment, the White test was used to assess homoscedasticity. In addition, tests on residuals, such as Cook's distance, DFBETAS, and DFFITS, were used to identify outliers and leverage points. The cutoff point for Cook's distance was set at the reference value of 4/*n*, where *n* represents the sample size. The variance inflation factor (VIF) was computed to assess multicollinearity, and values below 10 were considered acceptable. Several indices, such as the adjusted *R* ^2^, the Akaike and Bayesian information criteria, and the Bland‐Altman [34] limits of agreement, have been used to evaluate goodness of fit. In addition, Lin's concordance correlation coefficient (CCC), which incorporates Pearson correlation coefficient (a measure of precision) and the bias correction factor (a measure of accuracy), was used [35]. The Bland and Altman plots were used to visually assess agreement. The alpha level was set at 0.05 for each hypothesis test. Microsoft Excel (version 2024, Windows edition) was used to collect the dataset, while SAS (version 9.4, Windows edition) was used for statistical analysis.

## 3 Results

Twenty‐eight of the participants were female. Overall, 23.44% of swimmers were high‐ranked, while 17 participants were low‐ranked. The distribution of rank did not differ significantly by sex (*χ* ^2^_(1)_ = 0.1, *p* = 0.952), with no correlation between the categories (Spearman *ρ* = −0.039, 95% CI: −0.284, 0.206).

Table 1 reports the measurement consistency of the measurements, including accuracy (relative TEM), precision (ICC), and reliability. Skinfold thicknesses showed higher relative technical error (~3%) than BIA and ADP, associated with lower precision (~98.2%) and reliability (~99.4%).

**Table 1:** Internal consistency of the measurements.

Measure	TEM	*R*	Relative TEM	ICC	95% ICC	
Biceps SK	0.175	0.993	3.45%	0.986	0.978	0.991
Triceps SK	0.189	0.997	2.22%	0.994	0.990	0.996
Subscapular SK	0.180	0.991	2.30%	0.983	0.974	0.989
Suprailiac SK	0.212	0.994	2.21%	0.988	0.981	0.992
Supraspinal SK	0.219	0.993	3.35%	0.986	0.974	0.993
Abs SK	0.188	0.993	2.37%	0.985	0.976	0.991
Thigh SK	0.259	0.995	2.11%	0.989	0.984	0.993
Medial calf SK	0.223	0.996	2.54%	0.944	0.917	0.964
Lateral calf SK	0.534	0.967	5.60%	0.993	0.989	0.996
*R*	0.341	1.000	0.07%	0.999	0.999	1.000
Xc	0.221	0.999	0.36%	0.999	0.996	0.999
BV	0.099	0.999	0.17%	0.999	0.999	1.000

Abbreviations: BV, body volume; R, resistance; SK, skinfold thickness; Xc, reactance.

Overall, 93.63% of distributions met normality assumptions, while only 2.89% of comparisons showed significant differences.

Table 2 shows the descriptive statistics for female and male swimmers across their rank clusters. Overall, 32.5% of parameters showed a significant difference in female swimmers, especially in the high versus low comparison, compared with only 12.5% in males. Concerning BM and BV, coefficients of variation (CV) decreased linearly as the swimmers' rank increased in females (BM_mid_ = −9.28%, BM_high_ = −47.73%, BV_mid_ = −9.28%, BV_high_ = −47.3%), while the largest reduction was observed between low and mid ranks in males (BM = −30.06%, BV = −31.53%). The Lohman model showed the highest *d*_0_ mean value (1.095 ± 0.004), and significant differences were observed between males and females in all models (Brozek *F*_(2,62)_ = 55.34, *p* < 0.01; Lohman *F*_(2,62)_ = 35.21, *p* < 0.01; 2C‐Siri *F*_(2, 62)_ = 127.44, *p* < 0.01; 3C‐Siri *F*_(2,62)_ = 27.51, *p* < 0.01). Regarding anthropometry, stature (*F*_(3,61)_ = 1.42, *p* = 0.249), hip (*F*_(3,61)_ = 2.53, *p* = 0.088), and calf (*F*_(3,61)_ = 1.60, *p* = 0.21) girths did not show significant differences between ranks. In contrast, relaxed and stretched arm and thigh girths differed between low and high ranks (*p* < 0.05). Whilst no differences were observed between ranks in appendicular (*F*_(3,61)_ = 0.12, *p* = 0.889), leg (*F*_(3,61)_ = 0.29, *p* = 0.748), trunk (*F*_(3,61)_ = 0.66, *p* = 0.519) and total (*F*_(3,61)_ = 0.23, *p* = 0.792) skinfold thickness, females showed higher mean values than males in appendicular (40.2 vs. 28.45, *p* < 0.01), leg (24.01 vs. 18.01, *p* < 0.01), and total (66.64 vs. 53.89, *p* < 0.01) SKs; trunk averages were similar (25.18 vs. 25.44, *p* = 0.875). However, the sum of seven SKs (biceps, triceps, subscapular, suprailiac, abdominal, thigh, and medial calf) was 52.19 ± 10.46 and 74.73 ± 8.67 for high‐ranked males and females, respectively. As regards BIA, the phase angle differed between high and low males and females, while RH and XH showed significant differences only among high and lower rank male swimmers. Low‐ranked females showed a lower total body water content (*p* < 0.05).

**Table 2:** Summary statistics for female swimmers and two‐way ANCOVA comparisons adjusted by age.

	Female (*n* = 28)						Male (*n* = 36)						Two‐way ANCOVA					
	Rank: low (*n* = 7)		Rank: mid (*n* = 14)		Rank: high (*n* = 7)		Rank: low (*n* = 10)		Rank: mid (*n* = 18)		Rank: high (*n* = 8)		Sex		Rank		Sex#rank	
Variable	Mean	SD	Mean	SD	Mean	SD	Mean	SD	Mean	SD	Mean	SD	*F*_(1, 63)_	*p*	F _(2, 62)_	*p*	*F*_(2, 62)_	*p*
Age, year	13.98	0.77	14.88	2.74	21.47	3.67	17.39	3.88	18.50	3.35	22.24	5.81	—	—	—	—	—	—
Exp, year	9.98	2.05	9.52	3.83	16.61	3.88	12.64	3.59	13.56	3.96	16.87	4.23	0.1	0.758	0.58	0.562	0.71	0.497
Training, hour	13.00	1.55	13.25	0.85	15.00	2.24	13.25	0.86	13.53	2.58	14.00	2.24	0.86	0.358	0.37	0.689	0.39	0.679
BM, kg	49.80	7.36	53.64	7.19	61.90	4.78	70.18	12.49	67.13	8.35	75.26	10.67	32.99	< 0.01*	0.31	0.737	1.31	0.278
BV, L	47.48	7.22	51.19	7.06	58.95	4.72	65.94	11.96	62.81	7.80	70.82	9.91	26.73	< 0.01*	0.35	0.704	1.44	0.247
d, kg/L	1.049	0.012	1.049	0.009	1.05	0.003	1.064	0.009	1.069	0.01	1.063	0.008	53.25	< 0.01*	0.42	0.66	1.06	0.352
BF Siri 3C, %	11.44	2.56	12.48	2.45	14.42	1.71	8.96	3.59	8.06	2.32	9.78	4.23	67.25	< 0.01*	0.2	0.819	1.07	0.351
FFM Siri 3C, kg	43.96	5.43	46.76	5.16	52.91	3.06	63.53	9.19	61.61	6.91	67.76	8.60	73.06	< 0.01*	0.23	0.792	1.06	0.353
TBW, kg	28.02	3.95	30.1	3.87	34.36	2.48	46.01	6.72	43.95	4.85	49.41	6.48	133.86	< 0.01*	0.59	0.558	1.62	0.207
Stature, cm	160.8	7.39	164.08	5.12	169.61	6.50	175.47	5.01	178.21	6.94	179.69	8.28	47.85	< 0.01*	0.72	0.49	0.2	0.816
Arm relaxed, cm	25.51	2.07	26.29	2.43	29.26	1.00	30.29	3.76	29.29	2.37	31.89	3.32	13.25	< 0.01*	0.72	0.49	0.96	0.39
Arm stretched, cm	26.61	2.36	27.61	2.02	30.69	0.99	32.41	3.75	31.48	2.64	33.79	3.54	23.83	< 0.01*	0.44	0.647	1.09	0.343
Hip, cm	64.30	5.52	65.49	3.47	68.26	4.95	74.65	6.63	72.47	4.41	79.06	5.01	36.84	< 0.01*	0.9	0.413	2.7	0.076
Thigh, cm	47.34	5.04	48.54	4.16	52.60	1.82	52.59	5.23	51.48	3.69	55.46	4.36	3.4	0.07	0.58	0.561	0.61	0.549
Calf, cm	32.43	2.46	33.31	2.60	34.29	0.93	36.55	3.26	34.80	2.41	36.96	2.63	7.85	< 0.01*	0.8	0.543	2	0.144
Biceps SK, mm	5.98	1.54	6.18	2.04	5.74	1.78	4.18	1.55	3.6	1.11	3.54	1.23	27.9	< 0.01*	0.24	0.791	0.34	0.712
Triceps SK, mm	9.74	3.05	10.31	2.97	10.33	2.62	7.48	2.57	6.53	1.96	6.42	2.09	25.16	< 0.01*	0.09	0.91	0.59	0.559
Subscapular SK, mm	7.83	2.21	7.12	1.95	9.14	0.94	8.22	2.67	7.64	1.71	7.90	2.09	0.49	0.489	0.95	0.391	0.64	0.529
Suprailiac SK, mm	9.36	2.55	9.62	3.01	9.83	0.79	9.17	2.46	9.61	2.49	9.94	3.61	0.15	0.703	0.07	0.937	0.04	0.963
Supraspinal SK, mm	8.14	3.00	5.78	2.11	6.57	0.75	5.97	1.73	5.24	1.78	5.31	2.06	21.19	< 0.01*	0.06	0.939	4.94	0.011*
Abs SK, mm	8.08	0.83	7.21	2.63	9.47	1.43	8.23	2.63	7.63	2.04	8.65	2.62	0.17	0.686	1.11	0.339	0.22	0.807
Thigh SK, mm	12.33	4.67	13.26	3.66	16.98	1.92	11.27	3.09	10.94	2.95	8.88	1.76	21.19	< 0.01*	0.06	0.939	4.94	0.011*
Medial calf SK, mm	9.24	3.50	9.69	3.79	11.57	2.69	8.53	2.03	7.08	2.25	6.88	1.98	13.03	< 0.01*	0.45	0.64	1.96	0.151
Lateral calf SK, mm	9.36	3.03	9.85	3.19	10.98	2.36	9.35	2.42	9.07	2.36	8.38	3.11	2.08	0.155	0.01	0.994	0.92	0.405
Total SK, mm	60.58	13.02	66.15	16.20	71.42	11.23	57.08	13.58	52.87	11.08	52.19	10.46	17.03	< 0.01*	0.13	0.877	1.31	0.278
Appendicular SK, mm	37.29	11.37	39.44	10.88	44.62	6.89	31.47	7.75	27.99	6.73	25.71	5.56	26.18	< 0.01*	0.07	0.929	2.2	0.12
Leg SK, mm	21.57	7.85	22.95	7.01	28.55	4.09	19.80	4.56	18.03	4.69	15.75	3.26	20.13	< 0.01*	0.09	0.911	3.83	0.028*
Trunk SK, mm	25.08	4.17	23.89	6.56	27.17	4.29	25.62	7.21	24.88	4.96	26.48	7.67	0.01	0.965	0.28	0.758	0.04	0.959
Seven SK, mm	60.58	13.02	65.25	17.07	74.73	8.67	57.08	13.58	53.42	11.17	52.19	10.46	13.31	< 0.01*	0.05	0.947	1.36	0.265
PhA, °	6.37	0.43	6.08	0.41	6.91	0.50	6.90	0.53	7.08	0.53	7.78	0.98	18.18	< 0.01*	2.5	0.091	1.34	0.271
RH, Ω/m	370.65	39.22	354.8	45.69	324.19	20.56	255.36	30.91	277.69	36.68	237.81	34.9	69.2	< 0.01*	1.28	0.285	1.7	0.191
XH, Ω/m	41.77	2.93	38.03	3.68	39.76	3.07	31.02	2.77	34.84	4.65	32.47	2.56	38.8	< 0.01*	0.19	0.828	6.06	< 0.01*

*Note:* # Indicates interaction and * indicates statistically significant.

Abbreviations: BF, body fat; BM, body mass; BV, body volume; CFA, calf fat area; CFI, calf fat index; CMA, calf muscle area; d, body density; *n*, sample size; PhA, phase angle; R, resistance; RH, specific resistance; SD, standard deviation; SK, skinfold thickness; TBW, total body water; TCA, total calf area; TUA, total upper area; UFA, upper fat area; UFI, upper fat index; UMA, upper muscle area; Xc, capacitive reactance; XH, specific reactance.

Figure 1 shows the scatter plots and regression lines between the main BC parameters and BWA. Independent of sex, BWA was strongly correlated with age (*ρ* = 0.685, *p* < 0.001), BM (*ρ* = 0.545, *p* < 0.001), and stature (*ρ* = 0.499, *p* < 0.001), while it showed a weak correlation with body density (*ρ* = 0.175, *p* = 0.175). When stratified, females' BWA showed a strong correlation with arm girths (stretched *ρ* = 0.589, *p* = 0.001; relaxed *ρ* = 0.497, *p* = 0.007), whereas males' BWA showed the highest correlation with hip girth (*ρ* = 0.582, *p* < 0.001). Skinfold thicknesses showed no significant correlation with BWA, except for females' thigh SK (*ρ *= 0.4, *p* = 0.035). Concerning BIA, PhA shows a significant correlation in both sexes (Figure 1), and only RH was moderately correlated to BWA (*ρ* = −0.419, *p* = 0.026).

**Figure 1:**
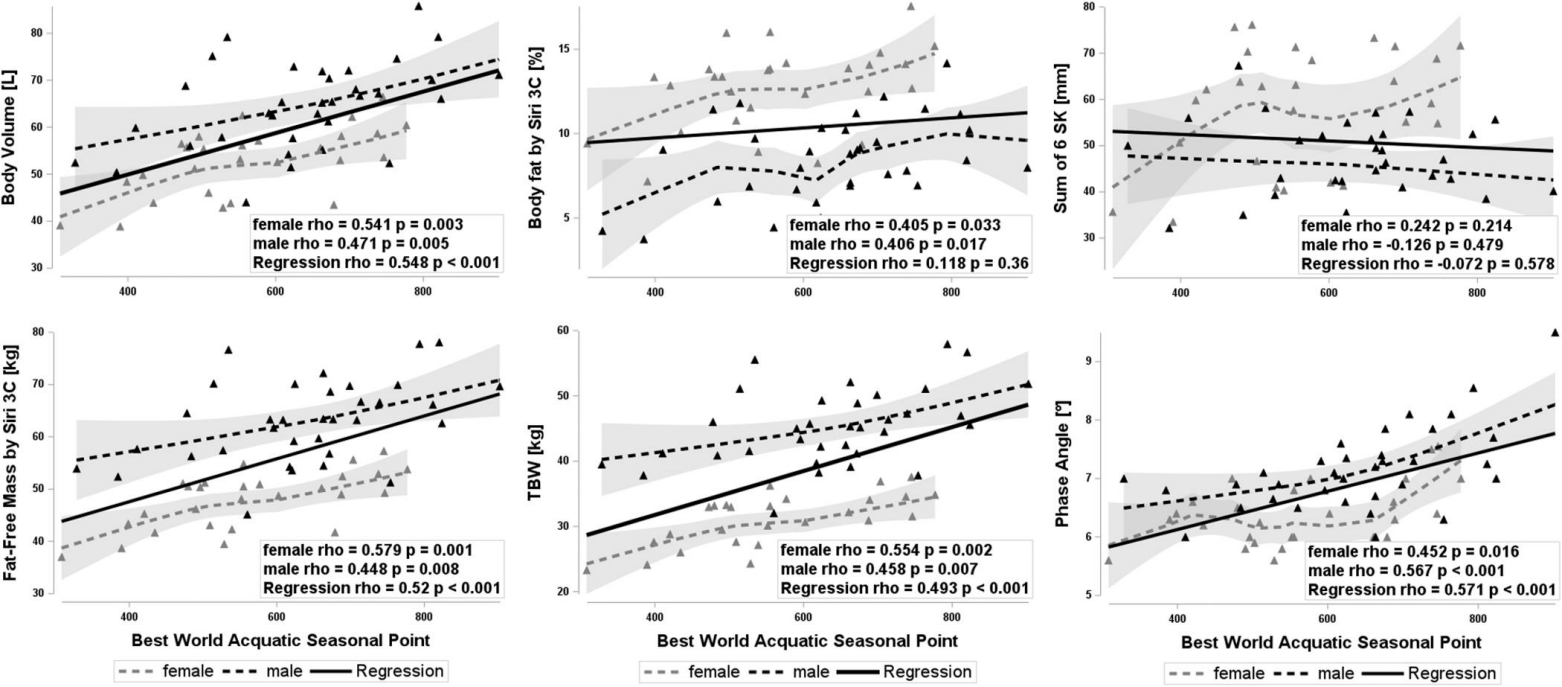
Scatter plots with regression line between BWA and body composition parameters, related to fat (top) and lean (bottom) traits. The grey dashed line and triangles represent female; the black dashed line and triangles represent male. The regression line is independent of sex. Abbreviations: *p*, p value; rho, Pearson correlation coefficient.

Given that 72.22% of significant differences were between high‐ranked and lower‐ranked females, and that these categories may be affected by age differences, a two‐way ANCOVA was further used to test the main parameters; the following description considers all the parameters adjusted for age. The stature showed no interaction effect (*F*_(2,63)_ = 0.20, *p* = 0.816), but showed a significant main sex effect (95% CI of *η* ^2^: 0.245, 0.571) with estimated values of 165.61 (95% CI: 163.18, 168.04) and 176.65 (95% CI: 174.42, 178.87) cm for females and males, respectively. Similarly, BM (95% CI of *η* ^2^: 0.161, 0.497), BV (95% CI of *η* ^2^: 0.122, 0.456) and *d* (95% CI of *η* ^2^: 0.273, 0.593) showed only significant main sex effects (*p* < 0.001), with estimated differences of 12.18 ± 2.09 kg, 10.59 ± 2.0 L and 0.017 ± 0.003 kg/L. Only the thigh SK showed significant interaction (95% CI of *η* ^2^: 0.11, 0.285) and main sex (95% CI of *η* ^2^: 0.083, 0.41) effects, whereas sex‐related differences in effect sizes ranged from *η* ^2^ = 0.296 (95% CI: 0.129, 0.464) at biceps to *η* ^2^ = 0.274 (95% CI: 0.111, 0.445) and *η* ^2^ = 0.158 (95% CI: 0.035, 0.334) at triceps and medial calf respectively, except for the subscapular (*F*_(6,63)_ = 1.66, *p* = 0.148), suprailiac (*F*_(6,63)_ = 0.21, *p* = 0.972), abdominal (*F*_(6,63)_ = 0.77, *p* = 0.601) and lateral calf (*F*_(6,63)_ = 0.67, *p* = 0.677) which showed no significant effects. Although supraspinal SK showed no significant interaction or main effect (*F*_(6,63)_ = 2.07, *p* = 0.072), the linear contrast of mid versus low (2.34 ± 0.96; *t* = 2.45, *p* = 0.018) and squared contrast of high versus low (3.56 ± 1.75; *t* = 2.03, *p* = 0.047) in females were significant. Concerning girths, only the main sex effect was significant, with different effect sizes at each body site: relaxed arm *η* ^2^ = 0.159 (95% CI: 0.036, 0.335), stretched arm *η* ^2^ = 0.256 (95% CI: 0.098, 0.428), hip *η* ^2^ = 0.352 (95% CI: 0.178, 0.513), thigh *η* ^2^ = 0.036 (95% CI: 0.00, 0.186) and calf *η* ^2^ = 0.097 (95% CI: 0.009, 0.266). However, hip also differed between high versus low (squared contrast = 6.85 ± 2.82, *t* = 2.43, *p* = 0.018) and high versus mid (linear contrast = 3.86 ± 1.86, *t* = 2.07, *p* = 0.043). Calf girth differed between high‐ and low‐ranked males (squared contrast = 3.20 ± 1.60, *t* = 2.0, *p* = 0.05) and between mid‐ and low‐ranked males (linear contrast = 2.05 ± 0.94, *t* = 2.19, *p* = 0.033). As regards BC, TBW showed a significant main sex differences (95% CI of *η* ^2^: 0.54, 0.765), as did body fat estimated by 2‐C Brozek (95% CI of *η* ^2^: 0.278, 0.596), 2‐C Siri (95% CI of *η* ^2^: 0.039, 0.342), 3‐C Siri (95% CI of *η* ^2^: 0.337, 0.639), 2‐C Lohman (95% CI of *η* ^2^: 0.171, 0.507), and FFM by 3‐C Siri (95% CI of *η* ^2^: 0.361, 0.655). Although the interaction effect of TBW/FFM was not significant, both main effects of sex (95% CI of *η* ^2^: 0.857, 0.931) and rank (95% CI of *η* ^2^: 0.0, 0.241) were significant. The estimated sex contrasts were 13.74 ± 1.2 (*t* = 11.41, *p* < 0.001), 6.87 ± 1.04 (*t* = 6.6, *p* < 0.001), 2.41 ± 0.83 (*t* = 2.91, *p* = 0.005), 4.75 ± 0.64 (*t* = 7.4, *p* < 0.001), 6.36 ± 1.21 (*t* = 5.26, *p* < 0.001) and 13.76 ± 1.63 (*t* = 8.43, *p* < 0.001), for TBW, 2‐C Brozek, 2‐C Siri, 3‐C Siri, 2‐C Lohman and FFM by 3‐C Siri, respectively. Differently, TBW/FFM showed an estimated linear sex contrast of 7.94 ± 0.33 (*t* = 21.03, *p* < 0.001), while rank differences were observed only in males, with two linear contrasts of 1.48 ± 0.52 (*t* = 2.83, *p* = 0.006) and 1.08 ± 0.46 (*t* = 2.34, *p* = 0.023) in high‐ versus mid‐ranked and mid‐ versus low‐ranked, and a squared contrast of 2.56 ± 0.79 (*t* = 3.24, *p* = 0.002) in high‐ versus low‐ranked. Finally, specific reactance (XH) showed interaction (*F*_(2,63)_ = 6.06, *p* = 0.004; *η* ^2^ = 0.137) and main sex (*F*_(1,63)_ = 36.38, *p* < 0.001; *η* ^2^ = 0.371) effects, while RH (95% CI of *η* ^2^: 0.346, 0.645) and PhA (95% CI of *η* ^2^: 0.067, 0.387) showed a significant main sex effect. However, high‐ and low‐ranked males estimates also differ in PhA (linear contrast = 0.55 ± 0.26, *t* = 2.11, *p* = 0.039) and RH (squared contrast = 53.10 ± 23.67, *t* = 2.24, *p* = 0.029), while XH showed the highest value in low‐ranked females (95% CI: 38.57, 44.48) and the lowest value in low‐ranked males (95% CI: 28.67, 33.32).

### 3.1 Body Composition Models

The newly proposed model I showed lower error propagation than model II (Table S1), with an average reduction of 6.42% (95% CI: 6.0, 6.8) compared with other models. It uses sex‐specific coefficients because rank did not significantly explain variability, providing two different equations:

(7) FemaleBF=((4.209∗BV)−(3.793∗BM))

(8) MalemidBF=((3.75∗BV)−(3.35∗BM))

The mean BF computed by the new model I was 12.08 ± 2.74% for males and 11.70 ± 2.92% for females, respectively. The 3‐C Siri model showed the highest mean value in females (12.71 ± 2.49%) and the lowest in males (8.70 ± 2.76%), while the 2‐C Lohman model showed the lowest mean in females (10.39 ± 3.05%). Although the new model I yielded the highest BF value in males, it showed the smallest variability (24.95%). Figure 2 shows scatter plots comparing previous models with the new models, with effect sizes (*η* ^2^) adjusted for sex and age. When adjusted, the new model I showed the closest agreement with the 2‐C Brozek BF (95% CI of *η* ^2^: 0.646, 0.82) with an RMSE of 0.319 (CV = 2.94%). The new model overestimates the mean BF by 1.01%, 2.05%, 0.97%, and 1.41%, respectively, compared with the 2‐C Brozek, Lohman, and Siri models and the 3‐C Siri model. However, when compared with other 2‐C models, error propagation of the new model I was reduced by 7.50 (95% CI: 7.09, 7.92), 4.52% (95% CI: 4.1, 4.94), and 4.05% (95% CI: 2.94, 5.17) compared with Brozek, Lohman, and Siri, respectively. In addition, relative error propagation decreases with increasing rank, with lower values in high‐rank females (2.42%) and males (3.05%).

**Figure 2:**
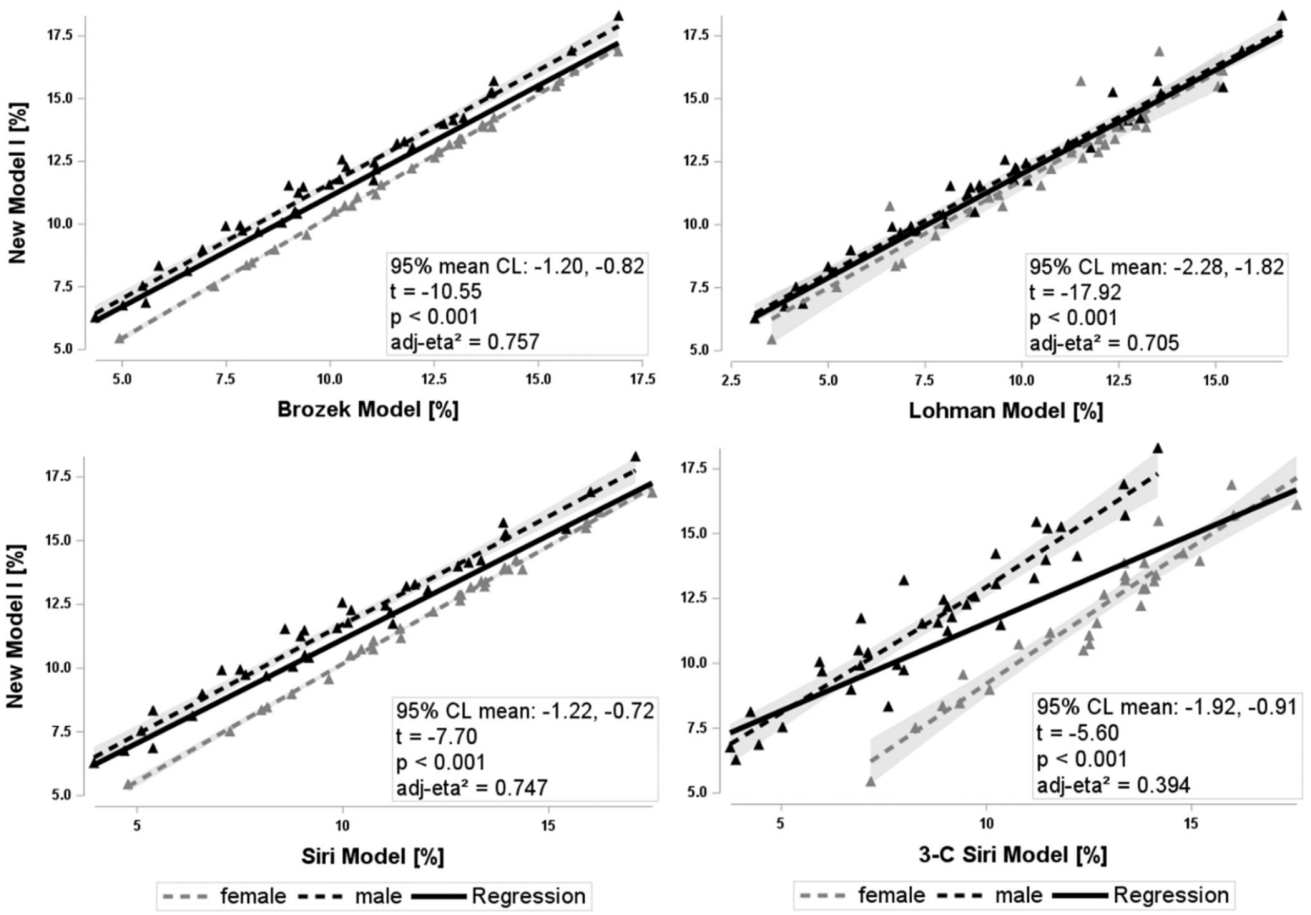
Scatter plots of body composition models and the new model I, with 95% of mean difference confidence limits (CL), student *t* statistics (*t*), and its *p* value (*p*), and the partial effect size adjusted by age and sex (adj‐eta^2^).

### 3.2 Body Volume Prediction Model

Pearson linear correlation coefficients showed the strongest associations between BV and girths, including stretched (*ρ* = 0.951, *p* < 0.01) and relaxed (*ρ* = 0.936, *p* < 0.01) arm, calf (*ρ* = 0.888, *p* < 0.01), thigh (*ρ* = 0.887, *p* < 0.01) and hip (*ρ* = 0.866, *p* < 0.01), as well as stature (*ρ* = 0.813, *p* < 0.01), while body density was strongly related to skinfold thicknesses at the biceps (*ρ* = −0.766, *p* < 0.01), triceps (*ρ* = −0.658, *p* < 0.01), thigh (*ρ* = −0.586, *p* < 0.01) and medial calf (*ρ* = −0.519, *p* < 0.01). When stratified by sex, BV showed the strongest correlation with thigh girth in females (*ρ* = 0.939, *p* < 0.01) and with stretched arm girth in males (*ρ* = 0.944, *p* < 0.01). The appendicular SK showed the strongest relationship with body density in both female (*ρ* = −0.534, *p* < 0.01) and male swimmers (*ρ* = −0.542, *p* < 0.01). Concerning BIA, BV was negatively related to both R (*ρ* = −0.785, *p* < 0.01) and Xc (*ρ* = −0.44, *p* < 0.01), and positively related to PhA (*ρ* = 0.694, *p* < 0.01); the magnitude of these relationships decreased for body density (R: *ρ* = −0.338, *p* < 0.01; PhA: *ρ* = 0.323, *p* < 0.01; Xc: *ρ* = −0.201, *p* = 0.111).

The bootstrapMA computation with the LASSO estimator under the AICC criterion showed the highest BIF values for stature (72%), stretched arm (96%), hip (66%), thigh (75%), and calf (77%) girths (Table S2). The selected model included six parameters and showed the highest selection frequency, equal to 18.81% (Table S3). The RMSD ratio was below 1 for all included regressors, indicating lower estimation error compared with the global model (Table S2). The relative conditional bias remained within approximately ±5%. The selected model showed comparable RMSE values in the training (*n *= 45) and test (*n *= 19) datasets, with a relative error difference of ~15% (Table S4). It showed an adjusted R^2^ = 0.981 (*F*_(5,63)_ = 587.93, *p* < 0.001) and an RMSE = 1.541 (CV = 2.61%). The Breusch–Pagan test indicated no evidence of heteroscedasticity (*χ* ^2^_(26) _= 32.63, *p* = 0.173), while the Durbin‐Watson test showed no evidence of autocorrelation (*D *= 1.954, *p*= 0.365). The mean VIF was 5.37. Figure 3 shows the agreement between observed and predicted BV and the Bland–Altman plot (bottom; 95% LoA: −5.541, 5.588). Lin's CCC = 0.984 (95% CI: 0.98, 0.993), with a bias correction factor (*C*_*b*_) = 0.999 and *r* = 0.987.

**Figure 3:**
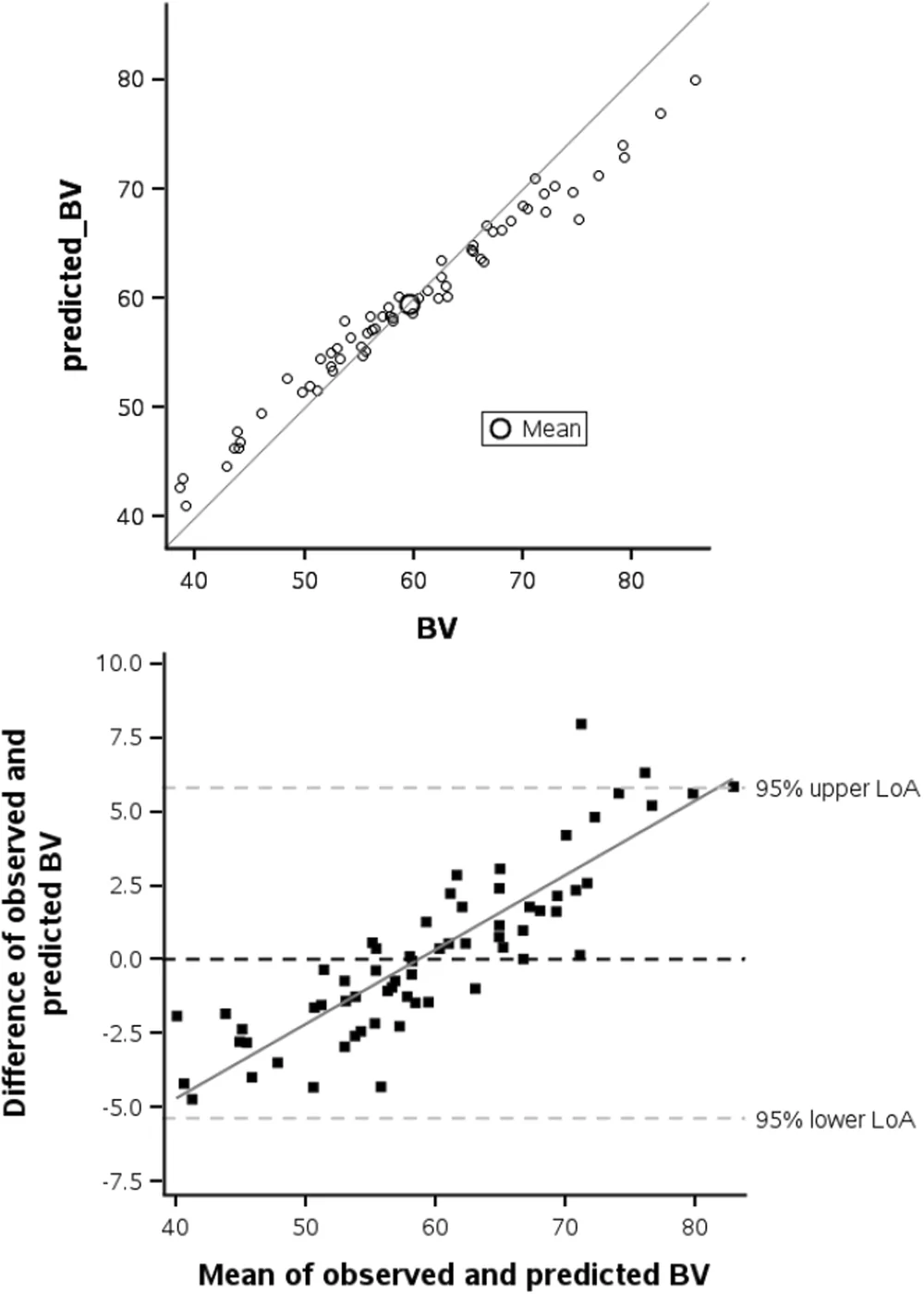
Agreement between the predicted BV by girths and the observed BV (ADP). At the top, the regression lines represent the predictability; at the bottom, the Bland–Altman plot. Abbreviation: LoA, limit of agreement.

The parameter estimates led to the following prediction model (Table S2):

(9) BV=−86.598+(0.342∗stature)+(0.905∗stretchedarm)+(0.117∗hip)+(0.659∗thigh)+(0.508∗calf)

where stature, stretched arm, hip, thigh and calf girths are measured in centimetres.

## 4 Discussion

This study aimed to examine differences in body traits according to rank and sex, assessed using anthropometric and BC techniques. As expected, we found that male and female swimmers showed different BC patterns, independent of rank. In contrast, chronological age may partly account for the apparent association between rank and BC, as several rank‐related differences were attenuated after adjustment for age. Although investigators still debate the appropriateness of standards and models in sports [36], our findings align with evidence suggesting that athletes may exhibit specific body features according to disciplines [14], sex [6], and rank within sport disciplines and sexes [37]. In our research, biological variability decreased by up to 47.7% as rank increased, with body densities similar to those of Olympic athletes (females 95% CI: 1.05, 1.06; males 95% CI: 1.07, 1.08 [14]). However, only a few parameters, such as BIA‐derived variables and hip girth, differed according to rank when comparisons were adjusted for age. This result suggests that sex, age and training volume may be important factors associated with swimmers' BC.

It is well established that sex plays a key role in defining body characteristics, but our findings further showed that females' BV, TBW, and FFM were respectively 16.41%, 30.31%, and 21.94% lower than those of males, whereas males showed lower BF values (4.75%). In addition, males exhibited larger arm (~9%) and hip (10.08%) girths and larger PhA values (8.66%), parameters strongly related to the best seasonal World Aquatics score. The association observed for PhA may reflect its relationship with neuromuscular properties relevant to physical performance [38]. However, when compared to other elite swimmers [6], high‐ranked females showed similar stature, but differed in arm (28.5–30.0 vs. 23.1–24.7) and thigh (51.3–53.9 vs. 43.1– 45.2) girths, and the sum of skinfold thicknesses (64.1–82.1 vs. 82.6–103.6), while high‐ranked males differed only in thigh girth (51.3–53.9 vs. 47.3–49.1). These discrepancies may be partly explained by a higher training volume (ranging from 10 to 15 h per week). Accordingly, as time spent in training decreased, the anthropometrical results converged to previous observations.

Regarding BC, our hypothesis was based on the relationship between human biological variability and sports‐specific physiological characteristics. The former may affect error propagation in body fat estimation [2], while the latter may contribute to long‐term physiological and morphological characteristics [12]. The anthropometric profiles were strongly correlated with BC estimates obtained using densitometric methods (hydrostatic weighing, ADP, DXA) and with skinfold thicknesses and girths; these parameters may therefore contribute to the assessment of fat and fat‐free mass compartments [39]. In addition, bioelectrical characteristics provide information on hydration status and body water content, which represent major components of lean body mass [40, 41]. We found that TBW accounted for 55.9% (95% CI: 55.8, 56.1) and 65.6% (95% CI: 65.5, 65.6) of body mass in female and male swimmers, respectively. Our findings were consistent with expected sex differences, with females showing lower total water content (95% CI: 54.7, 57.2) than males (95% CI: 63.3, 67.8). Although few studies have investigated water content in swimmers, Matias and colleagues (2016) found that male athletes had 29.1% higher water content than female athletes. In addition, males had higher FFM than females in both elite swimmers (+10.7%) and Olympians (+9.6% [6, 14]).

However, the previous estimates of fat‐free body proportions were obtained using general 2‐C or 3‐C models that did not account for specific sport‐ and sex‐related variability [13]. Siri [2] formulated a general mathematical model to describe BC from the whole‐body level to the atomic level, based on assumptions regarding tissue properties and component characteristics based on cadaveric dissections [29]. In the 2‐compartmental model, estimates may vary substantially depending on the density cut‐off points adopted, whether based on pure tissue values derived from cadaveric data or on restricted ranges obtained from specific samples. Thus, even when only two compartments are described, these differences may affect error propagation and introduce descriptive bias [13]. In light of this, we compared six BF estimation methods, two of which were developed to account for sample variability (new models I and II). The new model I defined body fat cut‐offs using the highest and lowest percentages for males and females, while the new model II assumed a fat fraction of zero within FFM, as proposed by Siri [2]. We found that BF varied from 0.2% to 49.5% within the same cluster. In addition, the first model had the lowest coefficient of variation among the other 2‐C models for females (−3.28%) and males (−8.5%), with an overall reduction in relative error propagation of ~2%. Thus, model selection appears to influence BF estimation [4], and sample‐specific approaches may reduce error propagation (Figure 2). Whilst the proposed method could introduce under‐ or overestimating of BF [22], the findings suggest the possibility of developing mathematical models that better account for athlete‐ and sport‐specific characteristics. This approach may represent a useful strategy for reducing biological variability and error propagation [13].

Evidence indicates that nuclear techniques have improved the characterization of molecular composition [4, 8], but there is still a need for daily assessments that can be performed using simpler and less expensive methods in practical settings. Nevertheless, the 4‐C model at the molecular level remains the “gold standard” for accounting for fat‐free components [42], yet it is still difficult to apply and requires expensive instruments such as DXA or mass spectrometry. By contrast, the 2‐C model, which distinguishes fat mass from fat‐free mass, requires the use of a plethysmograph or hydrostatic weighing to measure the subject's BV [28, 43]. The relationships among body mass, volume, and density enable us to estimate BF by selecting the preferred model, as previously discussed. In sports sciences, using ADP could be burdensome for many technicians, who may instead rely on anthropometry or BIA [44]. BV reflects the overall contribution of hydrophobic and water‐soluble tissue components and is therefore related to bioelectrical properties [45]. We found a strong negative relationship between BV and BIA raw parameters, indicating that fat‐free tissues account for a substantial proportion of BV in elite swimmers. About 70% of water is contained in striated muscles, which represent a major component of athletes' body mass [46]. The water proportions and the roles of ions in electrical conductivity justify the widespread use of BIA for estimating BC in athletes, with a focus on FFM [47].

However, clinicians might consider the greater variability of BIA compared with anthropometry for predicting BF [44]. To address this, we developed a new regression model to predict swimmers' BV from anthropometric measurements. Most previous prediction models used one or more skinfold thicknesses to estimate body density or fat, assuming a strong relationship between subcutaneous and total adipose tissue [5]. Although girth measurements show greater consistency than skinfold measurements [11], few models estimate BF using other anthropometric features [16], and only one study has used them to estimate BV in sports [13]. We used a bootstrap MA approach to select the most informative regressors, eliminate variables with negligible effect, quantify selection uncertainty, and obtain estimates. Whilst our global model did not meet the expected EPV (at least 130 participants [33]), the selected model showed encouraging internal stability. As expected, swimmers' girths, along with stature, explained approximately 98% of the variability in BV. In accordance with our initial hypothesis, girth measurements were among the strongest predictors of BV. Thigh and arm girths showed a strong linear relationship with BV (> 0.85 [13]), and their narrow variability across ageing may support the applicability of this approach across age [11, 17]. Some authors have evaluated the relationship between girths and BC parameters measured by DXA [48, 49, 50] and found strong correlations between limb girths and body fat or fat‐free mass. In addition, estimating BF or FFM without considering the mathematical model limits the assessment of error propagation. The prediction of BV may also be useful in contexts where BV or body density is required to construct multi‐compartment models alongside DXA or other techniques [51, 52].

This study has several limitations that should be considered and addressed in future studies: (i) BC was assessed according to densitometric theory using a 2‐compartment model. Multi‐compartment models may further reduce estimation error; (ii) retest reliability and external cross‐validation were not assessed; therefore, the external validity of the prediction model remains to be established. However, bootstrap MA was used to account for model uncertainty; (iii) participants were clustered according to their most recent best performance, while no additional performance tests were assessed; (iv) anthropometric and BIA assessments were performed according to specific international standards, although alternative instruments and guidelines are available. This may limit the generalizability of the proposed method and should be considered when applying it in different settings.

## 5 Practical Application

Both male and female swimmers may benefit from sex‐specific approaches to body composition assessment and performance monitoring. Even if BC shows limited variation across competitive ranks when international performance scores are considered, investigators need straightforward methods that can provide useful estimates for seasonal monitoring. Although advanced technologies allow increasingly detailed assessment of human body composition, sports science also requires practical and context‐specific methods. In accordance with the assumption that BC is estimated indirectly, measuring and predicting components strongly related to fat tissue, such as BV, may provide useful information. Deriving low‐cost, user‐friendly models with limited error propagation may support longitudinal monitoring in applied settings. The results of this study may help professionals to evaluate and monitor swimmers' health.

## Author Contributions

**Mario Mauro:** conceptualization, methodology, data curation, software, investigation, validation, formal analysis, visualization, writing – original draft. **Federica Moro:** conceptualization, data curation, investigation, software, methodology, validation, writing – original draft, visualization. **Vittorio Coloretti:** data curation, resources, validation, writing – review and editing. **Pietro Bosetto:** data curation, resources, validation, writing – review and editing. **Matteo Cortesi:** writing – review and editing, project administration, validation, supervision. **Stefania Toselli:** conceptualization, investigation, methodology, project administration, supervision, software, validation, writing – review and editing.

## Funding

The authors have nothing to report.

## Conflicts of Interest

The authors declare no conflicts of interest.

## Transparency Statement

The lead and corresponding authors (M. M. and F. M.) affirm that this manuscript is an honest, accurate, and transparent account of the study being reported; that no important aspects of the study have been omitted; and that any discrepancies from the study as planned have been explained.

## Supporting information


Supporting File 1

Supporting File 2

Supporting File 3

Supporting File 4

## Data Availability

The data that support the findings of this study are openly available in OSF at https://osf.io/g67uw/overview, reference number DOI: 10.17605/OSF.IO/G67UW.
