# Supplementary material for: Body Composition Differences Across Ranks and Sexes of Competitive Swimmers: A Cross‐Sectional Study
Source: Health Sci Rep. 2026 Oct 10;9(10):e73375. doi: 10.1002/hsr2.73375 (PMC13653321; doi:10.1002/hsr2.73375)
Supplement: Supplementary file 1 [file HSR2-9-e73375-s001.docx]

| **Table S1.** Error propagation for 2-C models | | | | | | | | | | | | | |
| --- | --- | --- | --- | --- | --- | --- | --- | --- | --- | --- | --- | --- | --- |
| sex | n | new mod I | new mod II | Brozek | Lohman | Siri | RRR:  I vs Brozek | RRR: I vs Lohman | RRR: I vs Siri | RRR: II vs Brozek | RRR: II vs Lohman | RRR: II vs Siri | RRR: I vs II |
| female | 28 | 4.99% | 7.07% | 8.44% | 6.80% | 8.97% | 6.30% | 3.30% | 7.26% | 2.51% | -0.49% | 3.47% | 3.80% |
| male | 36 | 4.20% | 10.51% | 10.27% | 8.20% | 4.79% | 8.70% | 5.73% | 0.85% | -0.35% | -3.31% | -8.20% | 9.05% |
