# Supplementary material for: Body Composition Differences Across Ranks and Sexes of Competitive Swimmers: A Cross‐Sectional Study
Source: Health Sci Rep. 2026 Oct 10;9(10):e73375. doi: 10.1002/hsr2.73375 (PMC13653321; doi:10.1002/hsr2.73375)
Supplement: Supplementary file 2 [file HSR2-9-e73375-s002.docx]

| **Table S3**. Model selection frequencies. | | | | |
| --- | --- | --- | --- | --- |
| Model | Included predictors | Count | Percent | n parameters |
| **1** | **Intercept stature arm_stret hip thigh calf** | **18** | **18.81** | **6** |
| 2 | Intercept stature arm_rel arm_stret hip thigh calf | 15 | 15.74 | 7 |
| 3 | Intercept arm_stret | 11 | 11.98 | 2 |
| 4 | Intercept arm_stret calf | 6 | 6.91 | 3 |
| 5 | Intercept stature arm_rel arm_stret hip thigh calf suprail_SK | 6 | 6.67 | 8 |
| 6 | Intercept arm_stret thigh | 4 | 4.9 | 3 |
| 7 | Intercept | 3 | 4 | 1 |
| 8 | Intercept stature arm_stret hip thigh calf suprail_SK | 3 | 3.72 | 7 |
| 9 | Intercept stature arm_stret | 2 | 2.89 | 3 |
| 10 | Intercept arm_stret thigh calf | 2 | 2.87 | 4 |
| 11 | Intercept stature arm_stret thigh calf | 2 | 2.84 | 5 |
| 12 | Intercept stature arm_stret hip thigh calf tric_SK | 2 | 2.71 | 7 |
| 13 | Intercept stature arm_stret hip thigh calf subs_SK | 2 | 2.7 | 7 |
| 14 | Intercept stature arm_rel arm_stret hip thigh calf tric_SK | 2 | 2.66 | 8 |
| 15 | Intercept stature arm_rel arm_stret hip thigh calf subs_SK | 2 | 2.66 | 8 |
| 16 | Intercept arm_stret hip | 1 | 1.87 | 3 |
| 17 | Intercept stature arm_stret calf | 1 | 1.86 | 4 |
| 18 | Intercept stature arm_stret thigh | 1 | 1.86 | 4 |
| 19 | Intercept arm_rel arm_stret calf | 1 | 1.77 | 4 |
| 20 | Intercept stature arm_stret thigh calf suprail_SK | 1 | 1.73 | 6 |
| Note: SK, skinfold thickness; n, number. | | | | |
