# Supplementary material for: Body Composition Differences Across Ranks and Sexes of Competitive Swimmers: A Cross‐Sectional Study
Source: Health Sci Rep. 2026 Oct 10;9(10):e73375. doi: 10.1002/hsr2.73375 (PMC13653321; doi:10.1002/hsr2.73375)
Supplement: Supplementary file 3 [file HSR2-9-e73375-s003.docx]

| **Table S2**. Global model, model selected by LASSO with AICC criterion and bootstrap-derived quantities for assessing model uncertainty. | | | | | | | | | | | |  |
| --- | --- | --- | --- | --- | --- | --- | --- | --- | --- | --- | --- | --- |
|  |  |  |  |  |  |  |  |  |  |  |  |  |
|  | **Global model** | |  | **Selected model** | |  |  |  |  |  |  |  |
|  |  |  | Bootstrap inclusion frequency (%) |  |  | Bootstrap inclusion frequency (%) | RMSD ratio | Relative conditional bias (%) |  | Bootstrap 5th percentile | Bootstrap 95th percentile |  |
| Predictors | Estimate | Standard Error |  | Estimate | Standard Error |  |  |  | Bootstrap median |  |  |  |
|  |  |  |  |  |  |  |  |  |  |  |  |  |
| Intercept | -54.452 | 6.394 | 100 | -86.598 | 1.769 | 100 | 0.28 |  | -86.284 | -100.188 | -70.961 |  |
| sex | -0.022 | 0.018 | 4 |  |  |  | 0.07 | 2.00 | 0 | 0 | 0 |  |
| stature | 0.238 | 0.020 | 72 | 0.342 | 0.012 | 72 | 0.29 | 2.12 | 0.364 | 0.222 | 0.42 |  |
| arm_rel | 0.133 | 0.032 | 33 |  |  |  | 0.23 | 3.93 | 0 | 0 | 0.182 |  |
| arm_stret | 0.826 | 0.050 | 96 | 0.905 | 0.031 | 96 | 0.09 | 1.16 | 0.898 | 0.658 | 1.115 |  |
| hip | 0.113 | 0.015 | 66 | 0.117 | 0.014 | 66 | 0.01 | 1.81 | 0.093 | 0.034 | 0.254 |  |
| thigh | 0.447 | 0.040 | 75 | 0.659 | 0.035 | 75 | 0.30 | 2.03 | 0.643 | 0.334 | 0.998 |  |
| calf | 0.376 | 0.036 | 77 | 0.508 | 0.046 | 77 | 0.21 | 1.82 | 0.527 | 0.097 | 0.898 |  |
| bic_SK | 0.015 | 0.010 | 4 |  |  |  | 0.09 | -1.61 | 0 | 0 | 0 |  |
| tric_SK | -0.022 | 0.009 | 11 |  |  |  | 0.14 | 1.80 | 0 | 0 | 0 |  |
| subs_SK | 0.009 | 0.007 | 7 |  |  |  | 0.07 | -0.94 | 0 | 0 | 0 |  |
| suprail_SK | 0.023 | 0.008 | 16 |  |  |  | 0.17 | -3.61 | 0 | 0 | 0 |  |
| thigh_SK | -0.004 | 0.003 | 4 |  |  |  | 0.07 | 0.42 | 0 | 0 | 0 |  |
| lat_calf_SK | 0.002 | 0.003 | 4 |  |  |  | 0.04 | -0.20 | 0 | 0 | 0 |  |
| Note: RMSD, root mean square difference; SK, skinfold thickness | | | | | | | | | | | |  |
