# Supplementary material for: Body Composition Differences Across Ranks and Sexes of Competitive Swimmers: A Cross‐Sectional Study
Source: Health Sci Rep. 2026 Oct 10;9(10):e73375. doi: 10.1002/hsr2.73375 (PMC13653321; doi:10.1002/hsr2.73375)
Supplement: Supplementary file 4 [file HSR2-9-e73375-s004.docx]

| **Table S4**. Bootstrap criteria summaries. | | | | | | |  |
| --- | --- | --- | --- | --- | --- | --- | --- |
|  | Bootstrap models with > 6 parameters | | Selected bootstrap model | | Other bootstrap models | |  |
|  |  |  |  |  |  |  |  |
| Criterion | Mean | SE | Mean | SE | Mean | SE |  |
| RMSE | 1.386 | 0.041 | 1.541 | 0.046 | 4.241 | 0.403 |  |
| Mean | 59.044 | 0.398 | 59.045 | 0.505 | 59.254 | 0.212 |  |
| R^2^ | 0.988 | 0.001 | 0.983 | 0.001 | 0.799 | 0.031 |  |
| Adj. R^2^ | 0.986 | 0.001 | 0.981 | 0.001 | 0.792 | 0.031 |  |
| AIC | 84.279 | 2.948 | 92.661 | 2.685 | 155.038 | 8.964 |  |
| AICC | 88.725 | 2.973 | 95.609 | 2.685 | 157.919 | 8.735 |  |
| SBC | 49.924 | 2.982 | 55.633 | 2.685 | 116.684 | 8.412 |  |
| ASE Train | 1.627 | 0.089 | 2.095 | 0.127 | 27.469 | 4.341 |  |
| ASE Test | 4.819 | 0.413 | 4.474 | 0.316 | 17.857 | 2.412 |  |
| Note: SE, standard error; RMSE, root mean square error; Adj., adjusted; AIC, Akaike information criterion; AICC, corrected Akaike information criterion; SBC, Bayesian information criterion; ASE train, average squared error training set; ASE test, average squared error test set. | | | | | | |  |
|  |  |  |  |  |  |  |  |
|  |  |  |  |  |  |  |  |
